# Study of the helminth fauna in booted eagle (*Hieraaetus pennatus*) in the South of Spain

**DOI:** 10.1016/j.ijppaw.2026.101249

**Published:** 2026-06-12

**Authors:** Pablo José Rufino-Moya, Ángela Salvador, Estefanía Jurado-Tarifa, Saúl Jiménez-Ruiz, David Cano-Terriza, Ignacio García-Bocanegra, Elena Zarco del Valle, Isabel Acosta García, Álvaro Martínez Moreno, Rafael Zafra Leva

**Affiliations:** a Unidad de Parasitología (ParaCOR), Departamento de Sanidad Animal, UIC Zoonosis y Enfermedades Emergentes ENZOEM, Universidad de Córdoba, Ctra. Madrid-Cádiz, km 396, Córdoba, 14071, Spain; b Instituto de Investigación e Innovación Biomédica de Cádiz (INIBICA), Hospital Universitario Puerta del Mar, Av. Ana de Viya, 21, Cádiz, 11009, Spain; c Departamento de Sanidad Animal, Grupo de Investigación en Sanidad Animal y Zoonosis (GISAZ), UIC Zoonosis y Enfermedades Emergentes ENZOEM, Universidad de Córdoba, Ctra. Madrid-Cádiz, km 396, Córdoba, 14071, Spain; d Grupo Sanidad y Biotecnología (SaBio), Instituto de Investigación en Recursos Cinegéticos IREC (UCLM-CSIC-JCCM), Universidad de Castilla-la Mancha, Rda. de Toledo, 12, Ciudad Real, 13005, Spain; e CIBERINFEC, ISCII CIBER de Enfermedades Infecciosas, Instituto de Salud Carlos III, Av. De Monforte de Lemos, 5, Fuencarral-El Pardo, Madrid, Spain; f Centro de Recuperación de Especies Amenazadas Quiebrajano (CREA Quiebrajano), Agencia de Medio Ambiente y Agua (AMAYA), Carretera Embalse Quiebrajano, km 15, Jaén, Spain

**Keywords:** *Hieraaetus pennatus*, Parasites, Helminthfauna, Ecological parameters

## Abstract

Studies on the helminth fauna of the booted eagle (*Hieraaetus pennatus*) remain limited, with only four parasitic species previously described and a single study reporting prevalence data. Accordingly, the objective of the present study was to characterize the helminth community parasitizing *H. pennatus* individuals from southern Spain (Andalusia). Between 2008 and 2023, a total of 28 specimens were examined. Recovered parasites were taxonomically identified, and prevalence, intensity, and mean abundance were calculated. The overall parasitism rate was 67.8%, comprising nematodes (53.5%), trematodes (21.4%), cestodes (25.0%), and acantocephalans (7.1%). Twelve genera of helminth were detected, eight of which are reported for the first time in this host. Among the eight helminths’ genera identified, six were fully identified and newly documented in *H. pennatus*. *Physaloptera alata* was the most prevalent species (25.0%), followed by *Capillaria tenuissima* (17.9%). Other detected taxa with lower prevalence (7.1%) included *Porrocaecum angusticolle*, *Platynosomum illiciens*, *Strigea falconis*, *Matabelea fuhrmanni*, and *Centrorhynchus buteonis*. The helminth community exhibited low diversity, with species richness of 1.6 (95% CI: 1.2-1.9), total abundance of 7.1 (95% CI:3.5-12.7), a Brillouin diversity index of 0.3 (95% CI: 0.09-0.35), and a Berger-Parker dominance index of 0.84 (95% CI: 0.74-0.95). These metrics reflect a depauperate parasitic community, with *P. alata* and *C. tenuissima* as dominant species that do not achieve the status of core taxa. This study represents the largest parasitological survey of helminths infecting booted eagles in Europe and the first conducted in southern Spain.

## Highlights

• Largest survey in booted eagles reveals a overal paratitation rate of 67.8%.
• Six especies fully identified for the first time in this host.
• Depauperate helminthfauna with low richness (1.6) and abundance (7.1).
• *P. alata* and *C. tenuissima* are the dominant species (0.84).

## Graphical abstract

**Figure:**
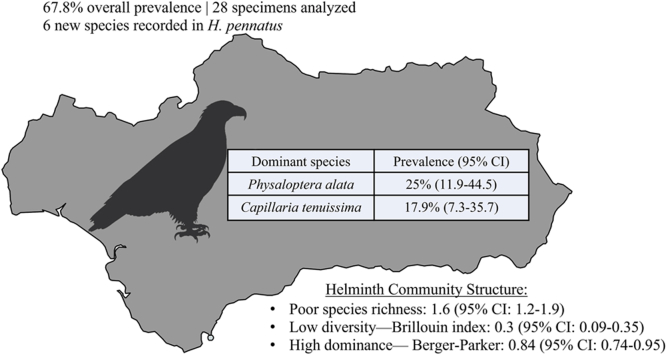

## 1 Introduction

Raptors occupy the apex of the trophic pyramid and are widely recognized as biologically significant, environmentally sensitive, and reliable indicators of ecosystem health (Bouchahdane et al., 2019). Consequently, research that enhances our understanding of raptors biology and the factors influencing their well-being is vital for maintaining ecological equilibrium. Given their pivotal role in foods webs, alterations in raptor health can trigger substantial cascading effects throughout the ecosystem (Illescas et al., 1993). As a predatory species, raptors serve as definitive hosts for numerous parasitic species transmitted through their prey (Sanmartín et al., 2004). In this context, parasitic organisms offer valuable insights into hosts ecology and environmental conditions and are frequently employed as biological indicators or ecological markers (Thomas et al., 1996). Furthermore, parasite assemblages have been used to explore the relationship between host productivity and species diversity across vertebrate taxa (Poulin et al., 2003).

The booted eagle (*Hieraaetus pennatus*) is a migratory raptor that breeds in Europe during the summer months, with individuals typically observed in Spain from March through September. Despite being predominantly associated with forest environments, this species exhibits marked ecological plasticity, occupying a wide range of habitats from coastal lowlands to mountainous regions at altitudes reaching 1600 m. Its diet is primarily avian, although it opportunistically incorporates reptiles and small mammals such as lagomorphs, with dietary composition varying geographically according to prey availability (García Dios, 2016). Major threats to this species include habitat degradation, direct persecution, and anthropogenic disturbance (BirdLife International, 2021). The European breeding population is estimated to range between 46,600 and 60,500 mature individuals, with Spain hosting the largest contingent -approximately 8000 breeding pairs-primarily distributed throughout the central-western regions of the Iberian Peninsula (García Dios, 2022). In Spain, documented causes of mortality include illegal shooting, collision with infrastructures or electrocution, as well as exposure to toxic substances such as rodenticides and pesticides (García Dios, 2016). This raptor is currently classified in Spain as a species of “Least Concern” (LC) (BirdLife International, 2021). Nevertheless, recent conservation assessments are expected to reclassify it as “Near Threatened” (NT) (SEO/BirdLife, 2025).

Both diurnal and nocturnal raptors are protected wildlife species, which poses considerable challenges for the acquisition and analysis of biological samples. These difficulties are compounded by the fact that samples are frequently obtained from individuals found deceased in the wild, often under suboptimal preservation conditions prior to examination. Such constraints severely limit the scope and reliability of diagnostic procedures —particularly in parasitological studies where morphological degradation and lack of sample integrity commonly prevent parasite identification at the species level (Zafra et al., 2022; Davitkov et al., 2026). As a result, comprehensive parasitological studies in raptors remain scarce, leaving notable gaps in our understanding of host-parasite interactions within these ecologically significant taxa.

Despite the ecological importance of birds of prey, the structure and composition of their parasite communities remain poorly understood. Recent studies show that helminths infecting Falconiformes, Accipitriformes and Strigiformes exhibit low inter-order similarity, with highly specific assemblages and minimal taxonomic overlap; indeed, Sitko and Heneberg (2019) reported that fewer than 40% of helminth species are shared across orders and that most taxa are strict specialists, underscoring the dominant role of host phylogeny in shaping these communities. This pronounced specificity, together with the authors’ evidence that helminth composition mirrors the deep evolutionary split between Australaves (Falconidae) and Afroaves (Accipitridae and Strigiformes), indicates that parasite distributions reflect not only contemporary ecological factors such as diet or habitat but also the evolutionary history of their hosts. Consequently, helminth communities in raptors represent a valuable framework for investigating coevolutionary processes, host-switching constraints and long-term ecological stability.

To date, only four published studies have investigated the helminth fauna of booted eagles (Anantaraman and Anantaraman, 1969; Illescas et al., 1993; Santoro et al., 2012a, Santoro et al., 2012b; Rentería-Solís et al., 2023), and among them, only Illescas et al. (1993) provides prevalence data. These studies are notably constrained by their small sample sizes, typically ranging from one to three individuals. The helminth species reported include *Centrorhynchus golvani* (India)*, Physaloptera alata* (Portugal)*, Synhimantus laticeps* and *Strigea falconis* (Italy). In Spain, only a single species from the genus *Physaloptera* has been recorded (Illescas et al., 1993). However, none of these studies offer comprehensive quantification of parasitism in raptors, as would be expected based on standardized metrics used in parasitological research on Accipitriformes and Falconiformes raptors (Sanmartín et al., 2004; Santoro et al., 2012a, Santoro et al., 2012b; Komorová et al., 2017). As a result, current knowledge regarding the helminth diversity in this raptor species remains highly limited.

The present study aims to characterize the helminth community in 28 booted eagle specimens from southern Spain (Andalusia) and to quantify parasitism using parameters that facilitate cross-comparative analysis with similar research conducted in other geographical regions.

## 2 Material and methods

### 2.1 Samples

A total of 28 booted eagles collected over a 15-years sampling period between 2008 and 2023 were examined for helminth parasites. Specimens originated from wildlife rescue centers in the Andalusian provinces of Cordova, Jaen, Cadiz, Seville and Malaga. The birds were either found dead in the wild or succumbed to injuries or illness during rehabilitation at these centers. Once received in the laboratory, they were identified and frozen at −20°C in plastic bags for subsequent necropsy.

The processing of the samples started with preliminary thawing (24 h at 4°C). Subsequently, the digestive tract (oesophagus, proventriculus, gizzard, and intestine), and the content was washed with saline and placed in settling cups. After that, both the digestive contents and the mucosal surface of the organs were inspected under a stereoscopic microscope for helminth identification. The same protocol was applied to the liver, gall bladder, heart, and lungs.

When helminths were detected, they were rinsed in saline and preserved in 70% ethanol. Specimens were then prepared for detailed morphological identification. Trematodes and cestodes were stained with Mayer's acid carmine and mounted in Canada balsam (Sigma-Aldrich®, Germany). Nematodes and acanthocephalans were cleared in Aman's lactophenol on glass slide, examined microscopically for identification, and subsequently returned to the preservative. Taxonomic identification keys were followed: Anderson (2000) for nematodes; Simon-Vicente et al. (2004), Gibson et al. (2002) and Bray et al. (2008) for trematodes; Khalil et al. (1994) for cestodes and, finally, Amin (2013) for acanthocephalans. Possible synonymies were assessed using the specialist-curated resource Fauna Europaea (https://portal.cybertaxonomy.org/fauna-europaea/).

### 2.2 Parasitological study

#### 2.2.1 Ecological parameters and biological index

To characterize the helminth community, ecological parameters were applied in accordance with the criteria established by Bush et al. (1997) to the booted eagles collected during the study period (2008-2023). Parasite species were further categorized into core, secondary, and satellite taxa following criteria of prevalence and abundance (Bush and Holmes, 1986). Thus, *core species* are those frequent (present in most hosts) and abundant; *secondary species* represent intermediate traits in terms of both distribution and population density within the host community; and *satellite species* are infrequently encountered and exhibit low abundance.

To assess structural attributes of the helminth community in the booted eagles studied, the following ecological metrics were applied: Species Richness (S); Total Abundance; Brillouin diversity index; and Berger-Parker dominance index.

#### 2.2.2 Statistical study

Descriptive parameters (prevalence, mean intensity, and mean abundance) were obtained using QPweb version 1.0.15 (Quantitative Parasitology on the web) (Reiczigel et al., 2019).

Past 5.2.2 (Paleontological Statistics) (Hammer et al., 2001) was used to calculate the biodiversity indices: Species richness, Total Abundance, Brillouin diversity, and Berger-Parker dominance.

Given the opportunistic nature and limited size of our dataset, we employed bootstrap resampling to generate conservative confidence intervals, acknowledging the inherent variability in our estimates. In addition, an accelerated bias-corrected bootstrap procedure with 2000 replicates was used to estimate confidence intervals (95% CI) for some descriptive parasitological parameters (intensity and abundance) as well as for biodiversity indices. Prevalence was calculated with a 95% confidence interval (95% CI) using Sterne's exact method.

## 3 Results

### 3.1 General results

Detailed table with all the parasitological data is available as supplementary material. The overall prevalence of helminth infection, classified by taxonomic groups was as follows: Helminths were detected in 67.8% (19 of 28) of the booted eagles examined. Nematodes were the most prevalent (53.5%; 15 of 28), followed by cestodes (25.0%; 7 of 28), trematodes (21.4%; 6 of 28), and finally, acantocephalans (7.1%; 2 of 28).

Table 1 shows the 12 identified helminth genera—six nematodes, three trematodes, two cestodes, and one acanthocephalan. In total, 140 helminths were recovered from the parasitized booted eagles. Of these, 117 (83.6%) were identified to species level. Unfortunately, species-level identification was not possible for the remaining 23 specimens (16.4%), which could only be assigned to genus level. This latter group comprised the following taxa: nematodes, including *Cyrnea* sp. (3.6%; n = 1), *Microtetrameres* sp. (3.6%; n = 1), *Physaloptera* sp. (3.6%; n = 1), *Synhimantus* sp. (7.1%; n = 2) and *Porrocaecum* sp. (3.6%; n = 1). A similar issue was encountered with trematodes: *Neodiplostomum* sp. (7.1%; n = 2) and *Strigea* sp. (3.6%; n = 1), and cestodes: *Cladotaenia* sp. (14,3%; n = 10). In addition, two cestode specimens recovered from a single individual could not be identified even at the genus level due to the advanced animal decomposition and were classified only at the broader taxonomic group.

**Table 1:** Helminths found in the 28 total booted eagles examined in the period 2008-2023.

Taxonomic Group	Eagles Parasitized	Species	n	P (95% CI)	I_M_	A_M_
Nematoda	15	*Physaloptera* sp.^a^	1	3.6% (1.9-17.4)	1^a^	0.04 (0-0.1)
		*Physaloptera alata*	17	25.0% (11.9-44.5)	2.43 (1.4-4)	0.61 (0.2-1.4)
		*Synhimantus* sp.^a^	2	7.1% (1.3-22.8)	1^a^	0.07 (0-0.2)
		*Synhimantus hamatus*	1	3.6% (1.9-17.4)	1^a^	0.04 (0-0.1)
		*Cyrnea* sp.^a^	1	3.6% (1.9-17.4)	1^a^	0.04 (0-0.1)
		*Capillaria tenuissima*	20	17.9% (7.3-35.7)	4 (1.6-9.8)	0.71 (0.2-2.2)
		*Porrocaecum* sp.^a^	1	3.6% (1.9-17.4)	1^a^	0.04 (0-0.1)
		*Porrocaecum angusticolle*	2	7.1% (1.3-22.8)	1^a^	0.07 (0-0.2)
		*Microtetrameres* sp.^a^	1	3.6% (1.9-17.4)	1^a^	0.04 (0-0.1)
Trematoda	6	*Strigea* sp.^a^	1	3.6% (1.9-17.4)	1^a^	0.04 (0-0.1)
		*Strigea falconis falconis*	8	7.1% (1.3-22.8)	4 (2-4)	0.29 (0-1)
		*Neodiplostomum* sp.^a^	4	7.1% (1.3-22.8)	2^b^	0.14 (0-0.4)
		*Platynosomum illiciens*	38	7.1% (1.3-22.8)	19 (13-19)	1.36 (0-4.1)
Cestoda	7	Unidentified^b^	2	3.6% (1.9-17.4)	2^a^	0.07 (0-0.2)
		*Cladotaenia* sp.^a^	10	14.3% (5.0-32.0)	2.5 (1-3)	0.36 (0.1-0.8)
		*Matabelea fuhrmanni*	16	7.1% (1.3-22.8)	8 (1-8)	0.57 (0-2.2)
Acantocephala	2	*Centrorhyncus buteonis*	15	7.1% (1.3-22.8)	7.5 (1-7.5)	0.54 (0-2.1)
		**TOTAL**	**140**			

n: number of parasites found; P%: Prevalence; I_M_: Mean intensity; A_M_: Mean abundance.

∗ only one infected host in the sample, CI cannot be calculated.

† Intensity is constant, bootstrap CI cannot be calculated.

a Not abl*e* to reach species level. b High decomposition

Eight helminth species were identified to species level: four nematodes, two trematodes, one cestode and one acanthocephalan. Considering the above, the most prevalent species were *P. alata* (25.0%; n = 17) followed by *Capillaria tenuissima* (17.9%; n = 20). The remaining species exhibited prevalences below 10%, grouped into two tiers: *Porrocaecum angusticolle*, *Platynosomum illiciens*, *S. falconis*, *Centrorhynchus buteonis* and *Matabelea fuhrmanni* were each detected in 7.1% of individuals, while *Synhimantus hamatus* was observed in 3.7%.

### 3.2 Helminth community structure

Of the 19 booted eagles parasitized, 10 showed co-infection involving two or more helminth species (six eagles with two species; two with three species; one with four species; and finally, one with five different species of helminths).

**Fig. 1:**
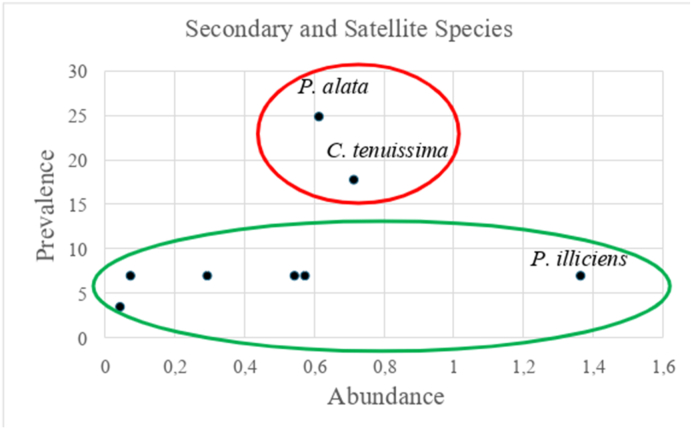
Distribution secondary (red) and satellite species (green).

According to the criteria by Bush and Holmes (1986) about the booted eagle's community, none of the helminth species in our study could be classified as core species (Fig. 1). However, two species were classified as secondary: *P. alata* (prevalence: 25.0%; mean abundance: 0.61) and *C. tenuissima* (prevalence: 17.9%; mean abundance: 0.71). All remaining helminths species (including *P. illiciens*, despite its relatively high abundance driven by marked intensity in a small number of infected hosts) were categorized as satellite species.

Community-level descriptors exhibited a species richness of 1.6 (95% CI: 1.2-1.9) and a total abundance of 7.1 parasites per host (95% CI: 3.5-12.7). Diversity metrics further characterized this community, with a Brillouin diversity index of 0.3 (95% CI: 0.09-0.35) and Berger-Parker dominance index of 0.84 (95% CI: 0.74-0.95).

## 4 Discussion

**Fig. 2:**
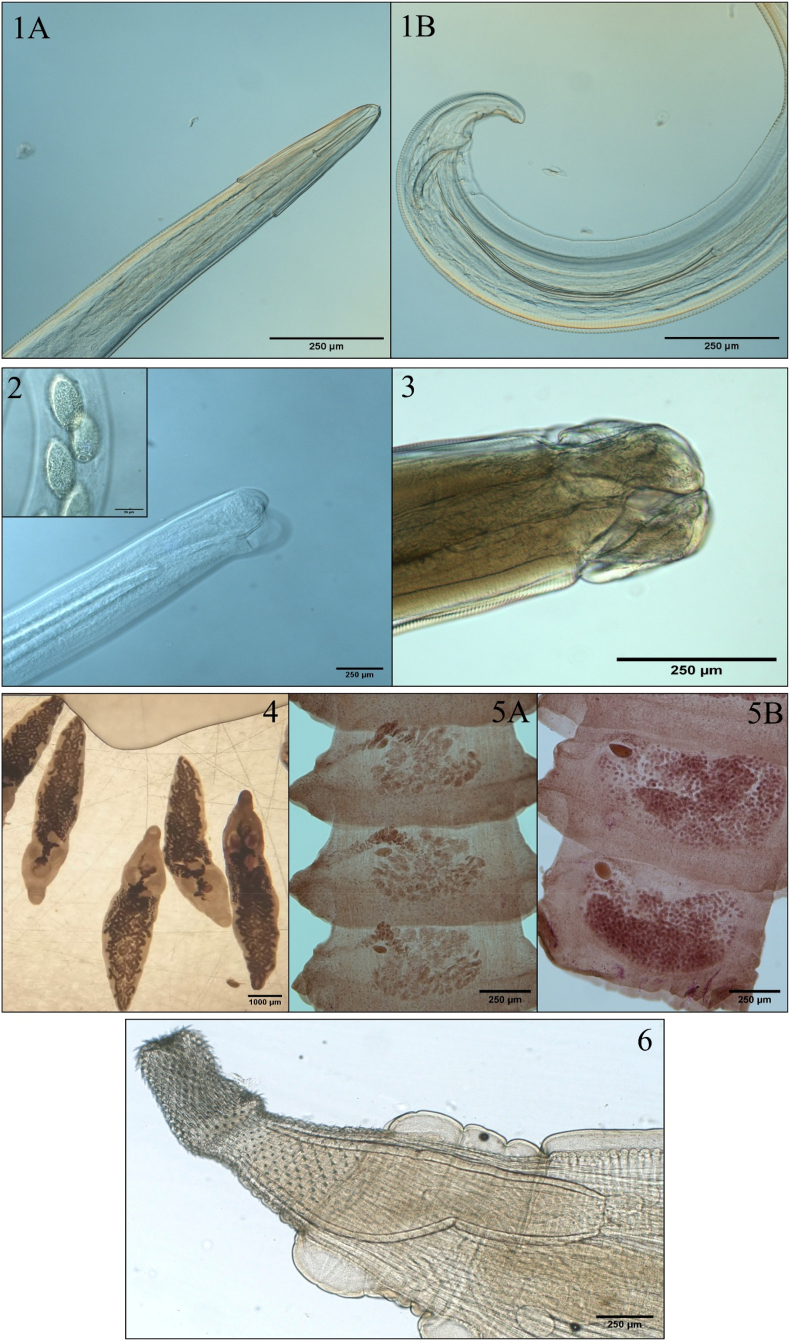
Photomicrographs of the six parasite species newly recorded in *H. pennatus*. 1: *S. hamatus* (male); 2: *C. tenuissima*; 3: *P. angusticolle*; 4: *P. illiciens*; 5: *M. fuhrmanni* (A: mature proglottid; B: gravid proglottid); 6: *C. buteonis*.

This study contributes to increasing the scarce data available on the helminth fauna of the booted eagle (*Hieraaetus pennatus*). Notably, eight genera (*Cyrnea*, *Capillaria*, *Porrocaecum*, *Microtetrameres*, *Platynosomum*, *Neodiplostomum*, *Cladotaenia*, *Matabelea*) and six species (Fig. 2: *S. hamatus*, *C. tenuissima*, *P. angusticolle*, *P. illiciens*, *M. fuhrmanni*, *C. buteonis*) are recorded for the first time in this host, expanding the known host range of several taxa (Illescas et al., 1993; Santoro et al., 2012a, Santoro et al., 2012b; Rentería-Solís et al., 2023).

The overall prevalence observed in our study (67.8%) is higher than the 33.3% previously reported for this species in Andalusia (Illescas et al., 1993). It is worth noting that this difference may be influenced by factors such as the larger sample size used in our study (28 individuals, compared to the 3 examined by Illescas et al., 1993), ecological changes in the environment, as well as the fact that this previous study is more than 30 years old. The prevalence we report also falls within the range documented for other European Accipitriformes, including 79.8% in northern Spain (Ferrer et al., 2004), 73.1% in Slovakia (Komorová et al., 2017), and up to 95% in Italy and 89.2% in Germany (Krone, 2000; Santoro et al., 2010).

The taxonomic pattern observed—nematodes as the dominant group, followed by cestodes, trematodes, and acanthocephalans—matches previous findings for Accipitriformes in Spain (Ferrer et al., 2004), Germany (Krone, 2000), and Slovakia (Komorová et al., 2017). However, prevalence values for several genera (*Capillaria*, *Synhimantus*, *Physaloptera*, *Microtetrameres*, *Porrocaecum*, *Neodiplostomum*, *Strigea* and *Cladotaenia*) were consistently lower than those reported for *Buteo buteo*, *Accipiter nisus*, and *A. gentilis* across Europe (Illescas et al., 1993; Sanmartín et al., 2004; Santoro et al., 2010; Komorová et al., 2017).

At a broader scale, the helminth community observed in *H. pennatus* aligns with the global patterns described for raptors (Sitko and Heneberg, 2019). These authors demonstrated that helminth assemblages in Falconiformes, Accipitriformes and Strigiformes exhibit consistently low similarity, with Sørensen indices typically below 0.40. This low overlap reflects strong phylogenetic structuring, where each order maintains a largely distinct parasite fauna despite ecological similarities. The species-poor and compositionally restricted community detected in *H. pennatus* fits within this framework, reinforcing the notion that Accipitriformes harbour helminth communities that are both taxonomically constrained and evolutionarily coherent.

This pattern is likely related to the trophic preferences of *H. pennatus*, as it is a raptor with a limited insectivorous diet. This is reflected, for example, in the prevalence of *P. alata*—the most prevalent species in this study yet only reaching 25%— which requires insects as intermediate hosts and amphibians or reptiles as paratenic hosts (Santoro et al., 2012a). The same hypothesis of a low insectivorous diet may also explain the prevalence of *Porrocaecum* and *Synhimantus* compared with other raptors (Ferrer et al., 2004; Sanmartín et al., 2004; Krone, 2000). A similar situation is observed for the second most prevalent species, *C. tenuissima* (17.1%), which requires the presence of earthworms or rodents for its transmission (Anderson, 2000; Atkinson et al., 2008; Zafra et al., 2022).

Trematodes such as *S. falconis* and *Neodiplostomum* spp. depend on aquatic snails and amphibians (Odening, 1966, 1967). The lower prevalence of these parasites in *H. pennatus* compared with *B. buteo* or *A. nisus* in Italy, Germany, and Slovakia (Krone, 2000; Santoro et al., 2010; Komorová et al., 2017) likely reflects reduced reliance on wetland habitats, which are critical for sustaining the intermediate hosts of these taxa. This hypothesis is corroborated by Krone and Jürgen Streich, 2000, who documented marked variations in *B. buteo* prevalence across three geographically distinct study areas in Germany, underscoring the influence of local habitat characteristics on parasite acquisition. However, trophic ecology alone does not fully account for the structure of helminth communities in raptors. Recent studies showed that most dominant helminths in birds of prey are not associated with specific prey categories, and that diet explains only a minor fraction of the variance in parasite distribution (Sitko and Heneberg, 2019). Instead, in this study the CCA analyses revealed a strong phylogenetic signal, with Accipitriformes, Falconiformes and Strigiformes each hosting distinct parasite assemblages. The helminth community of *H. pennatus*—dominated by taxa typically associated with Accipitriformes—supports this interpretation, suggesting that evolutionary history plays a central role in shaping parasite composition, beyond the constraints imposed by prey availability.

Cestodes such as *Cladotaenia* spp. rely on rodents as intermediate hosts (Santoro et al., 2010). The moderate prevalence observed here aligns with the relatively low mammal consumption of *H. pennatus* compared with *B. buteo* (García Dios, 2016). A special consideration deserves the other cestode species identified, *M. fuhrmanni*. This species it has been reported in African birds of prey such as *Aquila rapax*, *Circaetus cinereus*, *Falco biarmicus* and *Polemaetus bellicosus*, although unfortunately without quantitative data regarding prevalence to be compared (Southwell, 1925; Mettrick, 1963; Georgiev et al., 1994; Dimitrova et al., 2023). This cestode has not previously been recorded in *H. pennatus*, nor in any other diurnal raptor species used for comparative analysis in this study (*B. buteo*, *A. nisus*, *A. gentilis* or *Milvus* spp.). Thus, the finding of *M. fuhrmanni* in *H. pennatus* suggest a potential geographic and host specificity that has yet to be thoroughly investigated. The absence of prevalence data in African hosts, combined with its unexpected ocurrence in European raptors underscores the need for broader parasitological surveys and genetic analysis to confirm species identification and distribution patterns.

Finally, the low prevalence observed for *C. buteonis* is consistent with its heteroxenous life cycle, which involves arthropods and paratenic hosts such as reptiles and small mammals (Krone, 2000; Santoro et al., 2012a; Komorová et al., 2015, 2017). The prevalence rate reported in our study (7.1%) is similar to those found in other Spanish studies on Accipitriformes (Ferrer et al., 2004), although clearly lower than those reported in studies conducted in Italy and Slovakia (Santoro et al., 2010, 2012a; Komorová et al., 2015, 2017). There is no clear explanation for this discrepancy between Spain and other locations, although could be attributed to the limited exposure of *H. pennatus* populations in Spain to these specific intermediate and paratenic hosts.

Diversity metrics also differed markedly from those reported for other diurnal raptors. In southern Italy, species richness ranged from 2.85 to 4.54 and total abundance from 33.5 to 254.9 depending on host species (Santoro et al., 2012a), far exceeding the values observed in our study (1.6 for species richness and 7.1 for total abundance) for *H. pennatus*. Unlike other raptors, which typically exhibit a balanced distribution among core taxa such as *Centrorhynchus* in *B. buteo* and *F. tinnunculus*, or *Physaloptera alata* in *A. nisus* (Santoro et al., 2012a), the helminth community of *H. pennatus* is characterized by a marked dominance (Berger-Parker index: 0.84) of *P. alata* and *C. tenuissima*, which together constitute the vast majority of the parasite fauna in this host. These species with the highest prevalence (25% and 17.9%) and mean abundance (0.6 and 0.7, respectively) were numerically dominant within the helminth community. However, their relatively low prevalence and modest abundance indicate that they do not meet the criteria to be considered core species according to Bush & Holmes criteria (1986), for whom core taxa are characterized by both high prevalence and consistently high abundance across hosts. Empirical applications of this framework in parasitology typically identify core species only when prevalence exceeds approximately 40–50% and abundance remains elevated (Morand & Krasnov 2010). In addition, the high Berger–Parker dominance value (0.84) observed here should be interpreted with caution, as dominance metrics are known to be sensitive to small sample sizes and low-intensity infracommunities, where a few individuals belonging to one or two species can disproportionately inflate dominance estimates without reflecting true host-level ubiquity (Poulin, 1998; Magurran, 2004; Bush et al., 1997). Taken together, these considerations indicate that the dominance detected in this study reflects numerical aggregation rather than the presence of core species within the helminth fauna of this raptor. This highly skewed structure, reflected also in a low Brillouin index (0.3), suggests a depauperate community where ecological filtering has restricted establishment to a narrow subset of taxa. This pattern is consistent with previous observations by Sitko and Heneberg (2019), who reported that raptors with low helminth richness typically harbour simplified, uneven assemblages dominated by a few taxa—an outcome often linked to limited exposure to intermediate hosts or to phylogenetically constrained host–parasite associations. In *H. pennatus*, this reduced but apparently stable helminth fauna is closely tied to its trophic ecology: its predominantly avian diet and comparatively low consumption of insects and small mammals (García Dios, 2016) markedly limit encounters with intermediate hosts such as coleopterans, orthopterans, earthworms, and small mammals, which are required by many nematodes and cestodes (Anderson, 2000; Santoro et al., 2012a; Zafra et al., 2022). This restricted exposure provides a coherent explanation for the low species richness, limited diversity and numerical dominance of a few taxa observed in the community.

This study deals with several limitations (sample size, geographical restriction to Andalusia and opportunistic nature of carcass collection). In this sense, the bootstrap method and the CIs were neccesary to provide a more robust and conservative estimation of uncertainty inherent to small, non-parametric datasets. Also the identification based only on morphological features prevented species-level resolution for some taxa, suggesting certain over or subestimation of some parasitic species, underscoring the need for integrative approaches, i.e. morphological–molecular analyses (Santoro et al., 2012b; Rentería-Solís et al., 2023). Finally, the interpretation of our results must also consider the limited host metadata available for this study. Because carcasses were submitted opportunistically by Andalusian Wildlife Rehabilitation Centers (CREAs), only thoracic and abdominal organs were received, and the accompanying biological information was often incomplete. As a result, only the province of origin and the date of collection were consistently recorded (supplementary material), preventing any meaningful assessment of infection patterns in relation to host traits such as age, sex, body condition, or decomposition status.

Despite these limitations commented below, we believe that the present findings have significant implications for health and conservation of birds of prey. Raptors are widely recognized as bioindicators of ecosystem integrity (Santoro et al., 2012a; Zafra et al., 2022), and their helminth communities can reflect environmental conditions, prey availability, and habitat quality. As highlighted Sitko and Heneberg (2019), parasites with complex life cycles are particularly sensitive to habitat alteration and trophic simplification because they depend on multiple intermediate hosts whose abundance and distribution are easily disrupted by environmental change. The less of ecological complexity can therefore disproportionately affect specialist helminths, potentially leading to local parasite extinctions. In the case of *H. pennatus*, maintaining diverse and functional prey communities becomes essential not only for the conservation of the raptor itself but also for preserving the integrity of its associated parasite fauna, which in turn provides valuable information on ecosystem stability.

In conclusion, eight genera and six helminth species have been identified for the first time in this host. These findings indicate that the helminth community in *H. pennatus* is relatively species-poor and exhibits low biodiversity compared with others sympatric diurnal raptors. Within this community, *P. alata* and *C. tenuissima* emerge as the dominant species, since are present in most of the parasitized individuals. Given this raptor's obligate migration between European breeding grounds and African wintering sites, characterizing its helminth fauna is of epidemiological interest for this and other accipitriform raptors. Further research across additional geographical regions is required to evaluate spatial variation in helminth community composition for this raptor species and to assess potential ecological and biogeographical drivers influencing parasitic diversity. This study represents the first comprehensive analysis of the helminth fauna in the booted eagle (*H. pennatus*) in southern Spain (Andalusia), based on the largest sample size reported in Europe to date. Despite its limitations, this study provide the most extensive dataset currently available for this host species, contributing to the advance of the understanding of host-parasite interactions as well as a foundation for future ecological and epidemiological research.

## CRediT authorship contribution statement

**Pablo José Rufino-Moya:** Conceptualization, Data curation, Formal analysis, Investigation, Methodology, Supervision, Validation, Visualization, Writing – original draft, Writing – review & editing. **Ángela Salvador:** Data curation, Formal analysis, Investigation, Methodology, Writing – original draft, Writing – review & editing. **Estefanía Jurado-Tarifa:** Data curation, Formal analysis, Writing – review & editing. **Saúl Jiménez-Ruiz:** Writing – review & editing. **David Cano-Terriza:** Writing – review & editing. **Ignacio García-Bocanegra:** Funding acquisition, Resources, Writing – review & editing. **Elena Zarco del Valle:** Writing – review & editing. **Isabel Acosta García:** Conceptualization, Data curation, Formal analysis, Investigation, Methodology, Supervision, Validation, Visualization, Writing – original draft, Writing – review & editing. **Álvaro Martínez Moreno:** Investigation, Resources, Writing – review & editing. **Rafael Zafra Leva:** Conceptualization, Data curation, Formal analysis, Investigation, Methodology, Supervision, Validation, Visualization, Writing – original draft, Writing – review & editing.

## Declaration of competing interest

The authors declare that they have no financial, personal, or institutional conflicts of interest that could have influenced the research presented in this manuscript.
