# Supplementary material for: Study of the helminth fauna in booted eagle (Hieraaetus pennatus) in the South of Spain
Source: Int J Parasitol Parasites Wildl. 2026 Jun 12;30:101249. doi: 10.1016/j.ijppaw.2026.101249 (PMC13292471; doi:10.1016/j.ijppaw.2026.101249)
Supplement: Multimedia component 1 [file mmc1.pdf]

ID	Province	Date	*Physaloptera*	*P. alata*	*Synhimantus* sp.	*S. hamatus*	*Cyrnea* sp.	*C. tenuissima*	*Porrocaecum sp*	*P. angusticolle*	*Microtetrameres* sp.	*Strigea* sp.	*S. falconis*	*Neodiplostomum* sp.	*P. illiciens*	Cestodes (unidentified)	*Cladotaenia* sp.	*M. fuhrmanni*	*C. buteonis*		
1	Córdoba	19/04/2017	0	6	0	0	0	0	0	0	0	0	0	0	25	0	0	0	0		
2	Jaén	26/04/2017	0	1	0	0	0	0	0	0	0	0	0	0	0	0	0	0	0		
3	Jaén	14/05/2017	0	0	0	0	0	0	0	0	0	0	2	0	13	0	0	0	14		
4	Jaén	21/06/2017	0	0	0	0	0	0	0	0	0	0	0	0	0	0	0	0	0		
5	Jaén	22/06/2017	0	0	0	0	0	0	0	1	0	0	0	0	0	0	0	0	0		
6	Jaén	23/06/2017	0	0	0	0	0	0	0	0	0	0	0	0	0	0	0	0	0		
7	Jaén	29/06/2017	0	0	0	0	0	1	0	1	0	0	0	0	0	0	0	0	0		
8	Jaén	29/06/2017	0	0	0	0	0	0	0	0	0	0	0	0	0	0	3	0	0		
9	Córdoba	23/09/2017	0	0	0	0	0	1	0	0	0	0	0	2	0	0	0	0	0		
10	N.D.	05/10/2017	0	2	0	0	0	0	0	0	0	0	0	0	0	0	0	0	0		
11	Jaén	12/10/2017	0	1	0	0	1	0	0	0	0	0	0	2	0	0	1	0	0		
12	Jaén	20/10/2017	0	4	0	0	0	0	0	0	0	0	0	0	0	0	3	0	1		
13	Jaén	26/10/2017	0	0	0	0	0	0	0	0	0	0	0	0	0	0	3	0	0		
14	Jaén	10/09/2008	0	0	0	0	0	0	0	0	0	0	0	0	0	0	0	0	0		
15	Jaén	2013	1	0	0	0	0	0	0	0	0	0	0	0	0	0	0	0	0		
16	Jaén	2013	0	2	0	0	0	0	0	0	0	1	0	0	0	0	0	0	0		
17	Cádiz	02/04/2009	0	0	0	0	0	0	0	0	0	0	0	0	0	0	0	0	0		
18	Cádiz	11/09/2008	0	0	0	0	0	0	0	0	0	0	0	0	0	0	0	0	0		
19	Cádiz	08/06/2008	0	0	0	1	0	0	0	0	0	0	0	0	0	0	0	0	0		
20	Cádiz	06/06/2009	0	0	0	0	0	0	0	0	0	0	0	0	0	0	0	0	0		
21	Cádiz	16/03/2008	0	0	1	0	0	2	0	0	0	0	0	0	0	0	0	0	0		
22	N. D.	27/10/2017	0	0	0	0	0	0	0	0	0	0	0	0	0	0	0	0	0		
23	Málaga	07/07/2010	0	0	0	0	0	0	0	0	0	0	0	0	0	0	0	0	0		
24	Sevilla	19/02/2009	0	0	0	0	0	4	0	0	0	0	0	0	0	0	0	0	0		
25	Sevilla	10/09/2008	0	0	1	0	0	12	1	0	0	0	6	0	0	2	0	0	0		
26	Sevilla	16/09/2008	0	0	0	0	0	0	0	0	0	0	0	0	0	0	0	0	0		
27	Jaén	19/08/2023	0	0	0	0	0	0	0	0	0	0	0	0	0	0	0	15	0		
28	Jaén	01/04/2022	0	1	0	0	0	0	0	0	1	0	0	0	0	0	0	1	0		
**Total**			**1**	**17**	**2**	**1**	**1**	**20**	**1**	**2**	**1**	**1**	**8**	**4**	**38**	**2**	**10**	**16**	**15**	**140**	**100%**
**Identified**				**17**		**1**		**20**		**2**			**8**		**38**			**16**	**15**	**117**	**83.6%**
**Non identified**			**1**		**2**		**1**		**1**		**1**	**1**		**4**		**2**	**10**			**23**	**16.4%**

N. D.= Not determined
